# Biochar affects growth and shoot nitrogen in four crops for two soils

**DOI:** 10.1002/agg2.20067

**Published:** 2020-08-16

**Authors:** David Olszyk, Tamotsu Shiroyama, Jeffrey Novak, Keri Cantrell, Gilbert Sigua, Donald Watts, Mark G. Johnson

**Affiliations:** 1 Pacific Ecological Systems Division, USEPA, Center for Public Health and Environmental Assessment, 200 SW 35th St., Corvallis, OR 97333, USA; 2 Senior Environmental Employment Program, National Asian Pacific Center, 200 SW 35th St., Corvallis, OR 97333, USA; 3 USDA, Agricultural Research Service, Coastal Plains Soil, Water, and Plant Research Center, 2611 West Lucas St., Florence, SC 29501, USA

## Abstract

To address the need for information on biochar effects on crop growth and nitrogen (N), a greenhouse study was conducted with carrot, lettuce, soybean, and sweet corn using sandy loam (Coxville series) and loamy sand (Norfolk series) soils and a variety of biochars. Biochar was produced from pine chips (PC), poultry litter (PL), swine solids (SS), switchgrass (SG), and two blends of PC plus PL (50/50% [55] and 80/20% [82], wt/wt), with each feedstock pyrolyzed at 350, 500, or 700 °C. The results confirmed that biochar can increase crop growth; however, the responses varied with crop, soil, and feedstock and to a lesser extent with pyrolysis temperature. In general, lettuce had large increases in shoot and root dry weights vs. no-biochar controls with many biochars, primarily the SS and 55 blend and to a lesser extent with 82 followed by PL, and then PC and SG, especially when grown in the Coxville soil. Biochar had more limited effects on carrot, sweet corn, and soybean weights. Some biochars decreased crop growth (e.g., PL at 700 °C) for soybean shoot and pod dry weights with the Norfolk soil. Shoot N concentrations decreased with SS, 55, and 82 for carrot, lettuce, and sweet corn with the Norfolk soil but tended to increase for soybean. Shoot N uptake increased or decreased depending on biochar feedstock and temperature, crop, and soil. These results confirm that biochar can increase crop growth and affect shoot N, which is essential for crop growth.

## 1 | INTRODUCTION

Biochar, the residue following pyrolysis (heating in the absence of oxygen) of organic material (Lehmann & Joseph, 2009; 2015), has been the subject of intensive recent investigation as a solution to a variety of soil environmental problems. By altering soil pH (Joseph et al., 2010), fertility (Novak et al., 2009), structure and water relations (Atkinson, Fitzgerald, & Hipps, 2010), and soil microbes and mycorrhizae (Ennis, Evans, Islam, Ralebitso-Senior, & Senior, 2012; Warnok, Lehmann, Kuyper, & Rillig, 2007), biochar has the potential to increase crop productivity (Jeffery, Verheijen, van der Velde, & Bastos, 2011; Jeffery et al., 2017). Because biochar is relatively resistant to breakdown, it also enhances the carbon (C) sequestration capability of soils, thereby providing a mitigation option to reduce C-containing greenhouse gas concentrations in the atmosphere (Read, 2009).

Biochar can be produced from many different organic feedstocks from agriculture and forestry, including manures such as poultry litter (PL) (Chan, van Zwieten, Meszaros, Downie, & Joseph, 2008) and swine solids (Cantrell, Hunt, Uchimiya, Novak, & Ro, 2012) and wood or grass “wastes” (Novak, Cantrell, Watts, Busscher, & Johnson, 2014). Thus, production of biochar can help manage waste disposal problems (Lehmann & Joseph, 2009) by keeping them out of landfills and providing a beneficial use.

Although it has many potential beneficial uses, biochar alone is not a simple solution to alleviate all environmental problems and to concomitantly increase crop yield because results vary widely among biochar studies based on reviews and meta-analyses of data. Liu et al. (2013) and Jeffery et al. (2011) evaluated the effect of biochars on crop productivity (yield or aboveground biomass) and calculated 11 and 10%, respectively, increases in crop productivity across a variety of crops and experimental conditions. A wide range in changes (primarily increases) in crop biomass and yield also were found in reviews by Agegnehu, Srivastava, and Bird (2017), Ahmed, Kurian, and Raghavan (2016), Biederman and Harpole (2013), and Subedi, Bertora, Zavattaro, and Grignani (2017). Crop yield increases were found to be dependent on the soil. For example, Jeffery et al. (2017) indicated that biochar resulted in a 25% average increase in yield for acidic and low nutrient soils of the tropics. The meta-analyses by Jeffery et al. (2017) also emphasized that biochar did not increase yields in temperate areas. In terms of agricultural feedstocks, animal manures especially can result in improved soil fertility, which may enhance plant growth and yield (Liu et al., 2013).

The wide range of crop responses to biochar indicates that further research with a variety of crops is necessary to establish general patterns of response (e.g., increases in growth under similar biochar and soil conditions). A survey among vegetable crops in 2018 indicated that sweet corn and lettuce (all types) ranked first (200,158 ha) and third (118,209 ha), respectively, in terms of acreage planted in the United States (USDA, 2019a). Carrot is a major root vegetable crop grown on 32,699 ha in the United States (USDA, 2019a). Among major United States field crops, soybean was essentially tied for first with maize (field corn) at 36,096,341 ha planted in 2018 (USDA, 2019b). Soybeans also produce pods within a relatively short period of time that can be a useful reproductive endpoint for studies and are a legume depending on nitrogen (N) fixation for plant N requirements. Carrots, corn (as *Zea mays* L.), lettuce, and soybean are also recommended for use when testing the effects of pesticides and toxic chemicals on plant vegetative growth (USEPA, 2012).

The few reports specifically on biochar’s effects on sweet corn dry weights or yield have indicated varying results. For example, biochar made from an empty fruit bunch feedstock increased sweet corn shoot and root dry weights (Abdulrahman, Othman, Saud, & Abu Bakr, 2017), and empty fruit bunch biochar, and to a lesser extent wood or rice hull biochar, increased sweet corn shoot dry weight (Abdulrazzaq, Jol, Husni, & Abu-Bakr, 2015). Wood-based biochar at a high application rate decreased sweet corn shoot dry weight for a first crop with a loamy sand soil, but biochar at most rates increased sweet corn shoot dry weight for a second crop with both a loamy sand and a silty clay loam soil (Butnan, Deenik, Toomsan, Antal, & Vitykon, 2015). Tomato green waste biochar decreased sweet corn shoot dry matter due to adding salts to a sandy soil but increased shoot matter in a high-clay soil (Smider & Singh, 2014), and sugar maple biochar decreased sweet corn marketable yield, which was associated with a reduction in corn stalk nitrate-N (Cole et al., 2019).

In contrast to sweet corn, there have been many studies on field maize because of its importance as a crop in tropical to temperate areas. In meta-analyses, Bach, Wilske, and Breuer (2016) and Liu et al. (2013) estimated 7.5 and 8.4%, respectively, increases in maize crop productivity due to biochar application. Similarly, Jeffery et al. (2011) reported a minimal (<10%) increase in maize productivity with biochar application based on four studies (Gaskin et al., 2010; Kimetu et al., 2008; Major, Rondon, Molin, Riha, & Lehmann, 2010; Yamato, Okimori, Wibowo, Anshori, & Ogawa, 2006). In a more recent review, Jeffery, Abalos, Spokas, and Verheijen (2015) reported a mean increase in maize productivity of approximately 19% across a number of studies. In examples from specific studies, maize grain yield and/or biomass increased when biochar was added to fertilized fields (e.g., Agegnehu, Bass, Nelson, & Bird, 2016; Brantley, Savin, Brye, & Longer, 2015). In a few cases, grain yield decreased with biochar application without fertilization (Brantley et al., 2015), or biochar had no effect with or without fertilization (Unger & Killorn, 2011). The biochar effect on maize likely is concentration dependent: Gonzaga et al. (2017) reported that maize total biomass increased with biosolids biochar produced with a traditional kiln and applied at a rate 20 Mg ha^−1^ but decreased with 60 Mg ha^−1^. Rajkovich et al. (2012) reported a wide variety of maize biomass responses to biochar application depending on biochar feedstock, pyrolysis temperature, and feedstock application rate, ranging from a 43% increase with animal waste biochar to a 92% decrease with food waste biochar. Thus, despite the large number of studies, relationships are unclear among biochar feedstock type, pyrolysis temperature, soil type, and maize growth.

Soybean has had the most research of the three other crops proposed for this study. Jeffery et al. (2015) indicated a 22% mean increase in soybean productivity with biochar application across a variety of studies. For example, biochar increased soybean biomass for plants grown in an acidic Ferrosol and receiving biochar and fertilizer (Van Zwieten et al., 2010). Masud, Jiu-Ju, and Ren-Kou (2014) reported increased soybean biomass with the addition of canola straw or peanut straw (only at higher rate) biochar to an acidic Ultisol.

For lettuce, Jeffery et al. (2015) indicated a wide range of lettuce response to biochar with an average increase in productivity of just over 30%. In specific studies, Viger, Hancock, Migletta, and Taylor (2015) reported that poplar wood chip biochar and Gunes et al. (2014) found that poultry manure biochar increased shoot dry weight. Lettuce has been of interest as a test crop with biochar in developing countries. For example, Manka’abusi et al. (2019) reported that corn cob biochar increased lettuce shoot fresh weight for one of two cropping cycles in Burkina Faso. Steiner et al. (2018) reported that rice hull biochar increased lettuce fresh matter yield in a Ghana field study with urban farmers. Carter, Shackley, Sohi, Suy, and Haefele (2013) found that rice-husk biochar increased lettuce aboveground and belowground biomass across three growing cycles in a pot study in Cambodia. In contrast, other studies showed that there was no effect of biochar on lettuce leaf biomass, leaf N concentration, or N use efficiency in soil plus fertilizer (Pereira et al., 2015) or on lettuce fresh or dry weight (De Tender et al., 2016).

There have been few studies on the effects of biochar on carrots. Whole carrot plant (shoot and taproot) fresh weight and marketable yield were increased by the addition of corn cob biochar (Manka’abusi et al., 2019). However, carrot fresh weight was not affected by *Pinus radiata* pine woodchip biochar (Gartler, Robinson, Burton, & Clucas, 2013). George, Kohler, and Rillig (2016) found that pine wood, pine bark, wood pellet, and spelt husk biochars did not affect carrot shoot or fine root biomass. For taproots, pine wood and pine bark biochars also did not affect biomass, whereas wood pellet or spelt husk biochars increased biomass (George et al., 2016).

Nitrogen is essential for plants and is a key part of many critical metabolites (Hawkesford et al., 2012). The effects of biochar on plant N concentrations have been studied in several crops and there have been several reviews that consider N. Biederman and Harpole (2013) conducted a meta-analysis, which indicated that biochar when applied across a variety of crops generally had no effect on plant tissue N concentration. In a review describing the relationship between biochar and soil and plant N, Clough, Condron, Kammann, and Müller (2013) reported that biochar resulted in a range of responses, such as no effect or a decreased foliar N content but increased plant N uptake. In an example from a specific study, maize leaf N concentration may be increased following the application of biochar (Agegnehu et al., 2016). In contrast, Rajkovich et al. (2012) reported an overall general decrease in maize total plant N concentration and total N uptake with increasing pyrolysis temperature and increasing biochar application rate across a variety of feedstocks, with exceptions (e.g., N uptake increasing at the lowest temperature with PL biochar). For soybean, Masud et al. (2014) reported increases in shoot N uptake with canola straw at 10 or 20 g kg^−1^ or peanut straw at 20 g kg^−1^ biochar application. Lettuce shoot N increased with application of poultry manure biochar (Gunes et al., 2014).

Thus, although there is considerable literature on the effects of biochar on crop growth, to make better recommendations for biochar use, a synthesis of information relating to biochar type, soil type, and crop type is needed (Jeffery et al., 2015). In terms of crop N, data on biochar and nutrients are especially needed to develop guidelines for use of biochar that are coordinated with soil fertilization and nutrient management planning (Gul & Whalen, 2016). Therefore, the objectives of this study were to fill these knowledge gaps by (a) determining the effects of a variety of different biochars on crop productivity using four important crops grown under similar greenhouse conditions, (b) determining the effects of soil type on biochar responses, and (c) determining not only growth responses but also effects on N, a major plant nutrient.

## 2 | MATERIALS AND METHODS

### 2.1 | Soils and biochars

To compare crop responses to biochar with different soils, two agricultural soils were obtained from a cultivated field at the Clemson University Pee Dee Research and Education Center Farm in the Coastal Plain region of South Carolina. One soil was a Norfolk Series (fine-loamy, kaolinitic, thermic Typic Kandiudults [loamy sand]), and the other was a Coxville Series (fine, kaolinitic, thermic Typic Paleaquults [fine sandy loam]). Both soils are highly weathered Ultisols, with the Norfolk series being well drained and the Coxville series being poorly drained. The uppermost horizon (~0–23 cm) of both soil profiles was use in this study. Site cultivation history, crop management, and additional characteristics of the soils are described elsewhere (Novak et al., 2014; Olszyk, Shiroyama, Novak, & Johnson, 2018; Olszyk et al., 2020; Sigua et al., 2014) and are summarized in Table 1.

After collection, both soils were air-dried and sieved. The Norfolk soil was sieved to pass a 2-mm sieve. The Coxville soil was sieved to pass a 4-mm sieve because it is a heavier-texture soil, having higher clay and silt contents. Because the state of South Carolina is under a soil quarantine due to imported fire ants (*Solenopsis invicta* Buren), the USDA Animal and Plant Health Inspection Service regulations required that the South Carolina soils be frozen to −23 to −29 °C for at least 24 h prior to shipping them to Oregon.

Biochars were produced in South Carolina by pyrolysis at 350, 500, or 700 °C and held at the high temperature for 1–2 h. The biochars were made from a variety of agricultural and forestry feedstocks: switchgrass straw (*Panicum virgatum*) (SG), loblolly pine chips (*Pinus taeda*) (PC), swine solids (SS), and PL. Pure materials of SG, PC, SS, and PL and two mixtures of PC and PL were used as well as mixtures of 50% PC and 50% PL (55) and 80% PC and 20% PL (82) by weight. The blending ratios for the biochar mixtures were made based on their P release dynamics as outlined in Novak et al. (2014).

Full details of the biochar production conditions can be found in Novak, Cantrell, and Watts (2013) Novak et al. (2014), Olszyk et al. (2018, 2020), and Sigua et al. (2014). Each feedstock was air dried, ground by passing through a Wiley mill with a 6-mm screen (PL and SS) or hammer milled (SG and PC), and made into pellets. For mixtures, PC and PL feedstocks were combined prior to pelletization. Pellets were made with a mill (PP220, Pellet Pros, Inc.) with a 6-mm flat die and roller set (Cantrell, Martin, & Novak, 2014). Pellets of each feedstock were then pyrolyzed at a low (350 °C), medium (500 °C), and high (700 °C) temperature (furnace-retort system, Lindberg/MPH) for 1–2 h (Cantrell et al., 2014) based on sample size. Prior to chemical analysis, all biochars were ground to pass through a 0.25-mm sieve and stored in a desiccator.

Key chemical characteristics of the biochars used in this study are shown in Table 2. Data are based on Novak et al. (2013, 2014), Olszyk et al. (2018, 2020), and J. Novak (personal communication, 2016, 2020). The nutrients were measured by a commercial laboratory (Bureau Veritas Minerals) (Olszyk et al., 2020).

The soil and biochar (pellets and pellet fragments) treatments were prepared following the methods described in Novak et al. (2014). In brief, to obtain a 1% mixture (~20 Mg ha^−1^) of biochar in soil by weight, for soybean and sweet corn a target of 950 g of air-dried soil was weighed into a plastic sealable bag, and then a target of 9.5 g of biochar was added. The level of 1% biochar was chosen because it represents a realistic field application rate and is similar the rate used in the field with crops such as maize (Gaskin et al., 2010; Major et al., 2010). For lettuce and carrots, a target of 450 g of soil was weighed into a bag, and then a target of 4.5 g of biochar was added. Each bag was then thoroughly mixed by hand and spread out onto plastic coated butcher paper. On the basis of personal communications with J. Novak, the following procedure for initially wetting the soil was recommended. Deionized water at an amount to obtain a soil moisture content of 10% (wt/wt) was added to the soil (with and without biochar), and then water and soil were gently mixed using a trowel. The moist soil was quantitatively transferred to clean plastic pots with geotextile cloth (to limit soil loss from the bottom of the pots) lining the bottom of the pot.

### 2.2 | Crop growth conditions

This study used four crops: carrot [*Daucus carota* subsp. *sativus* (Hoffm.) Schübl. ‘Tendersweet’], lettuce (*Lactuca sativa* L. ‘Black-Seeded Simpson’), soybean [*Glycine max* (L.) Merr. ‘Viking 2265’], and sweet corn (*Zea mays* L. ‘Golden Bantam’). Seeds from the respective crop plants were planted in the appropriate green plastic pots for plant size: 15.2 cm diameter (soybean and sweetcorn) or 10.2 cm diameter (lettuce and carrot). For each respective treatment, three seeds of sweet corn and soybean and approximately eight lettuce and five carrot seeds were planted per pot. Soybean seeds were inoculated with Guard-N rhizobia bacteria (Currently Verdesian Life Sciences) prior to planting. Dates of planting, emergence, and harvest are shown in Table 3. Just after planting, lettuce, soybean and sweet corn received KH_2_PO_4_ equivalent to 67 kg ha^−1^ P and 85 kg ha^−1^ K in soil. Lettuce and sweet corn also received NH_4_NO_3_ equivalent to 112 kg ha^−1^ N in soil. The fertilizer was dissolved in reverse-osmosis (RO) water, and a pipettor was used to deliver the target amount of N, P, and K to the pots. Slightly more fertilizer was added to the carrot pots due to a slight drift in the pipettor setting (i.e., 72–74 kg ha^−1^ P, 92–94 kg ha^−1^ K, and 121–124 kg ha^−1^ N in soil). During plant growth, RO water was applied whenever the top of the soil was dry to the touch. Plants were thinned to one plant per pot after germination by culling the largest and smallest plants per pot. If there were two seedlings per pot, one was randomly removed. If there was any remaining uncertainty as to which plant should be removed, the other pots in the treatment were observed to determine if an unusual plant should be removed. Plants were grown in a greenhouse under 1,000 W high-intensity discharge lights with a 12 h light/12 h dark photoperiod for soybean and sweet corn and with a 16 h light/8 h dark photoperiod for lettuce and carrot. Average daily greenhouse environmental conditions from emergence to harvest are shown in Table 3.

### 2.3 | Crop measurements

Crop growth was determined primarily as dry weight at harvest. For all crops, shoots were cut off at the soil surface, dried, and weighed. Lettuce, carrot, and corn plants were essentially all leaf material because these were young, vegetative plants, whereas soybean shoots contained a small amount of stem material. For soybean, pods were removed separately from the plants and counted prior to drying and weighing. Drying was at 60 °C, except for lettuce and carrot shoots, which were dried at approximately ambient air temperature for 29 and 22 d, respectively, prior to drying at 60 °C. For roots, prior to harvest pots for each crops were randomly assigned to two groups of three pots each: Group A included those for which the root systems were to be immediately obtained, and Group B included those for which the pots were put into cool storage at 4 °C until they were leached with RO water and the leachate collected for pH and soil nutrient quantification (data not presented). For Group A pots, root systems were washed with RO water to remove the soil, followed by drying at 60 °C and weighing. After leaching, root systems from Group B pots were processed following the same procedures as described for Group A. Carrot roots were divided into taproot and diffuse (fine) root components prior to drying.

Dried shoot samples from all crops were submitted to the USDA laboratory in Florence, SC, for elemental analysis. Leaf samples were ground using a Wiley mill. A TruSpec CN analyzer (LECO Corp.) was used to measure total organic C and total N. The shoot N concentration was reported on a g kg^−1^ basis, and N uptake was reported on a g shoot^−1^ dry weight basis, which was calculated as shoot N concentration times shoot dry weight. For elemental (K and P) analysis, samples were digested using automated block digestors according to USEPA method 3050B (USEPA, 1996). The digestate was analyzed for total elemental concentrations using inductively coupled plasma –optical emission spectroscopy (Vista-PRO [Varian] or Optima 8300 [PerkinElmer]). Elemental concentrations were expressed on a mg g^−1^ dry shoot weight basis.

### 2.4 | Experimental design and statistical analysis

For each crop there were biochar control plants and 18 biochar treatments (6 feedstocks × 3 pyrolysis temperatures); these 19 treatments were repeated for each soil type. There were six replicates per each of the 19 treatments and two soil types, for a total of 228 pots planted per crop. The study followed a completely randomized design. Pots were randomly located across a greenhouse bench and rotated to change position at least once during the experiment in case any environmental gradient had developed in the greenhouse over time. In addition, later during the study, half the corn plants were transferred to a second bench to provide more space for final growth. Even though half the corn plants were on the second bench, the pots were assumed to be randomly located.

Prior to statistical analysis, plant dry weight and N data were log transformed because treatment effects were assumed to be additive on a log scale. If necessary due to “0” values, a small value (one order of magnitude smaller than smallest value) was added prior to the log transformation of the data. Pod number was square root transformed to achieve homogeneity of the variance. However, because of the heterogenetity of variance, for all parameters for each species a weighted ANOVA was used, with weights proportional to the inverse of the variances for the transformed data for each soil and treatment (Welch, 1951).

Data were analyzed separately for each crop using the PROC MIXED ANOVA procedures in SAS/STAT software (SAS Institute, 2013): Version 9.4 of the SAS System for Windows. To determine interactions among soil and biochar treatments, the control plants were not included, and the ANOVA factors were soil type (Norfolk and Coxville), temperature (350, 500, and 700 °C), and feedstock (PL, SS, SG, PC, 55, and 82). A separate ANOVA was carried out to compare all treatment effects, including the control treatment (i.e., not biochar) plants. The factors were soil and treatment (18 soil and/or biochar treatments plus control plants) and soil × treatment interactions. A Dunnett’s test was used to compare individual treatments with the control plants at *p* < .05 for each soil type. Means and SEs in the figures are approximations based on back transformations of the least square means and SEs (average based on upper and lower least square SEs) obtained from the statistical analysis.

## 3 | RESULTS

### 3.1 | Soil and biochar characteristics

The finer-textured loamy sand Coxville soil had a lower pH, higher K and P concentrations, and much higher organic C than the coarser-textured sandy loam Norfolk soil (Table 1) (Olszyk et al., 2018, 2020; Sigua et al., 2014). The Norfolk soil N concentration was below the level of detection. The PL, 55, and 82 biochars had similar but higher pH levels than the SS, PC, and SG biochars (Table 2) (Olszyk et al., 2018, 2020). The SS biochar had the highest N, Ca, Mg, and P concentrations, followed by the PL, 55, and 82 biochars, in descending order. The PL biochars had the highest K and Na concentrations, followed by the 55, SS, and 82 biochars. The PC and SG biochars had low concentrations of all elements. Increasing the pyrolysis temperature from 350 to 500 or 700 °C greatly decreased the H and O concentrations and, to a lesser extent, extractable P concentrations of biochars (except for SG) but had less effect on other elemental concentrations.

### 3.2 | Crop growth

Feedstock type was the most important factor affecting plant growth, with significant effects at <.001 according to the ANOVA for all crops and response parameters across soils and temperatures except for carrot shoot dry weight (Table 4). Soil type also had a major effect on plant growth, with significant effects for nearly all crops and parameters except carrot taproot and lettuce shoot dry weights. In contrast, pyrolysis temperature had much less effect on plant growth than feedstock type, with only one significant response to temperature alone (sweet corn shoot dry weight). Generally, the feedstock response differed with soil type, as indicated by the many significant feedstock × soil interactions. There were some feedstock × temperature and feedstock × temperature × soil interactions but only two temperature × soil interactions (carrot and sweet corn shoot dry weights). Because of the soil and treatment interactions, our focus was on responses to individual feedstock × temperature treatments vs. the soil-only controls (no biochar) using Dunnett’s test separately for each crop and soil.

**FIGURE 1:**
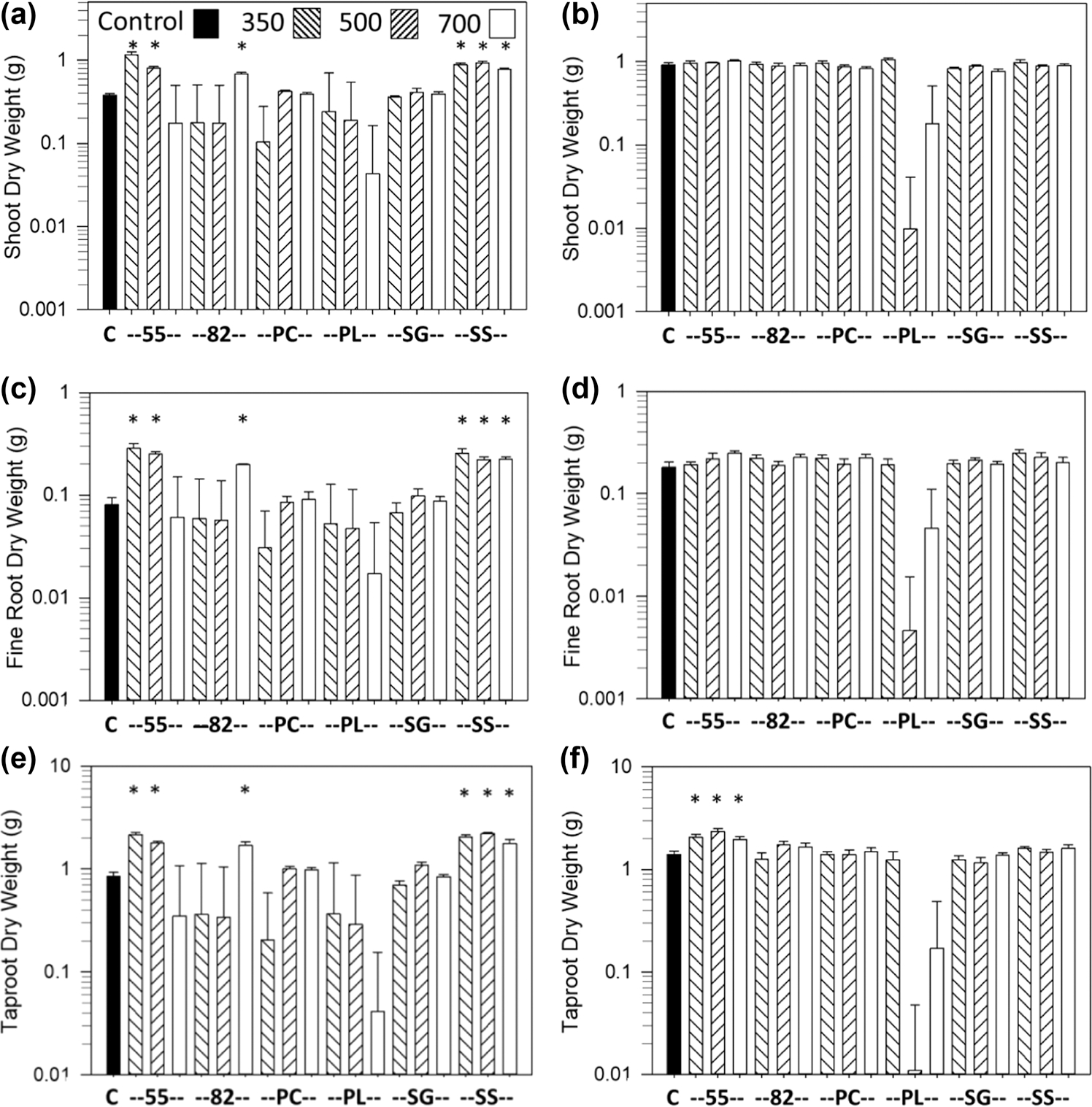
Effects of biochar on carrot dry weight. Data are for shoot dry weight for (a) Norfolk or (b) Coxville soil, fine root dry weight for (c) Norfolk or (d) Coxville soil, and taproot dry weight for (e) Norfolk or (f) Coxville soil. Pyrolysis temperatures (°C) are indicated at the top of graph (a). Each bar represents the average plus the unpooled upper average SE for six pots. An asterisk above a bar indicates a significant difference vs. control plants according to Dunnett’s test. There were no significant biochar effects on shoot or fine root dry weight for the Coxville soil. C, no biochar control; PC, pine chips; PL, poultry litter; SG, switchgrass; SS, swine solids; 55, 50% PC and 50% PL; 82, 80% PC and 20% PL.

**FIGURE 2:**
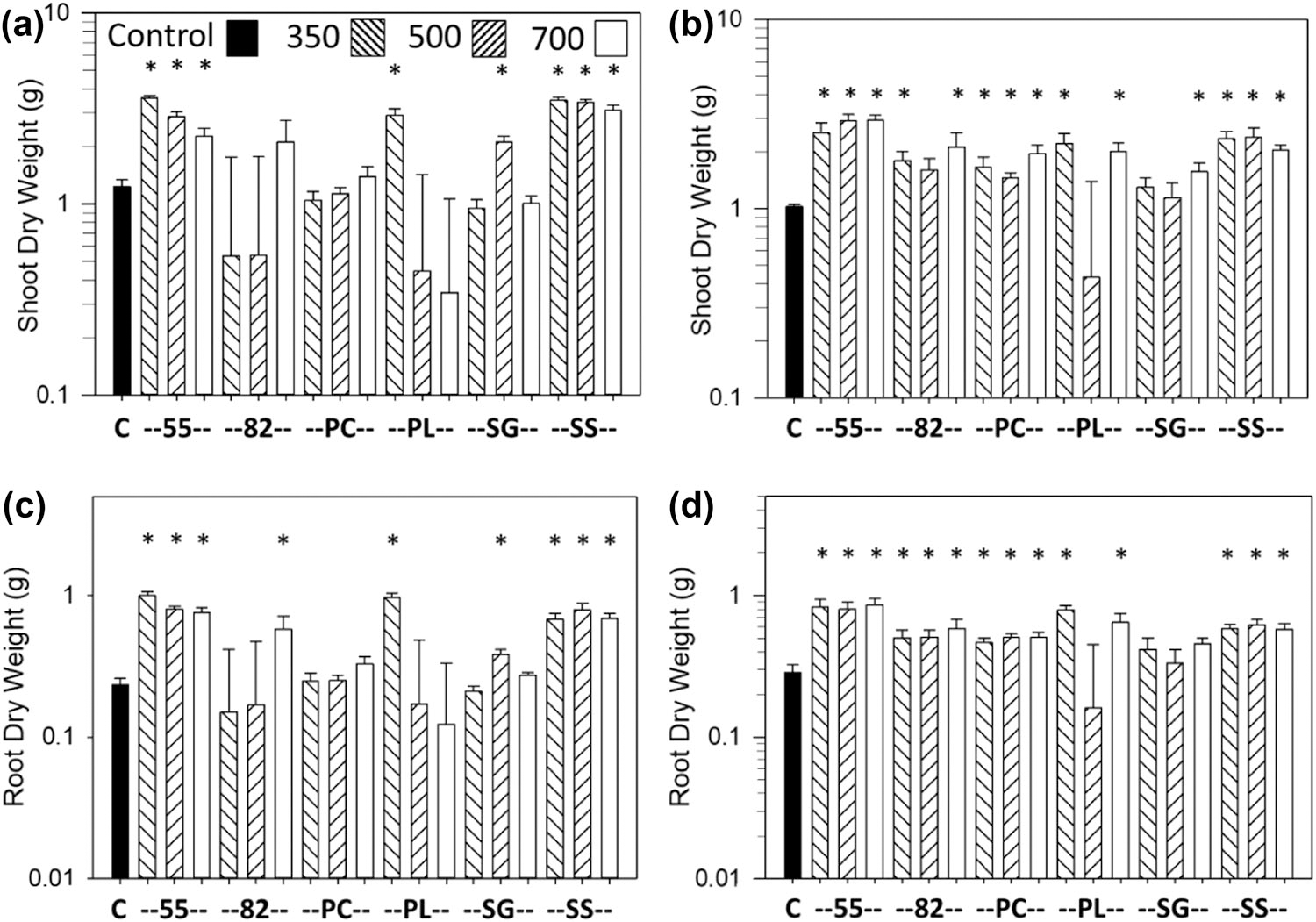
Effects of biochar on lettuce dry weight. Data are for shoot dry weight for (a) Norfolk or (b) Coxville soil and root dry weight for (c) Norfolk or (d) Coxville soil. Pyrolysis temperatures (°C) are indicated at top of graph (a). Each bar represents the average plus the unpooled upper average SE for six pots, except for five pots for the shoot dry weight and Norfolk PC 350 and root dry weight for the Coxville SG 350 and Norfolk PC 350 treatments. An asterisk above a bar indicates a significant difference vs. control plants according to Dunnett’s test. C, no biochar control; PC, pine chips; PL, poultry litter; SG, switchgrass; SS, swine solids; 55, 50% PC and 50% PL; 82, 80% PC and 20% PL.

**FIGURE 3:**
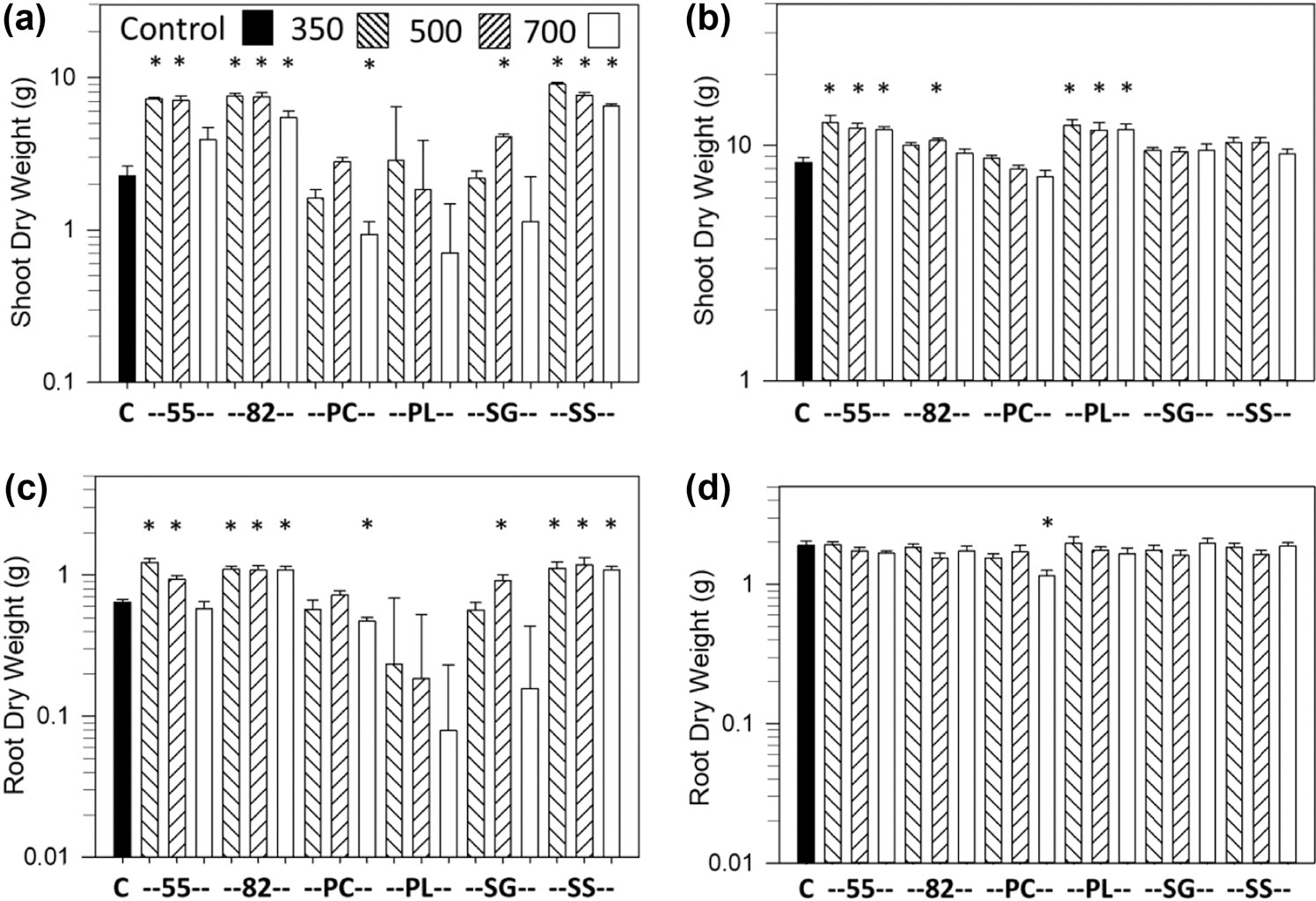
Effects of biochar on sweet corn dry weight. Data are for shoot dry weight for (a) Norfolk or (b) Coxville soil and root dry weight for (c) Norfolk or (d) Coxville soil. Pyrolysis temperatures (°C) are indicated at top of graph (a). Each bar represents the average plus unpooled upper average SE for six pots, except for five pots for the shoot dry weight Norfolk soil control treatment. An asterisk above a bar indicates a significant difference vs. control plants according to Dunnett’s test. There were no significant biochar effects on root dry weight for the Coxville soil. C, no biochar control; PC, pine chips; PL, poultry litter; SG, switchgrass; SS, swine solids; 55, 50% PC and 50% PL; 82, 80% PC and 20% PL.

**FIGURE 4:**
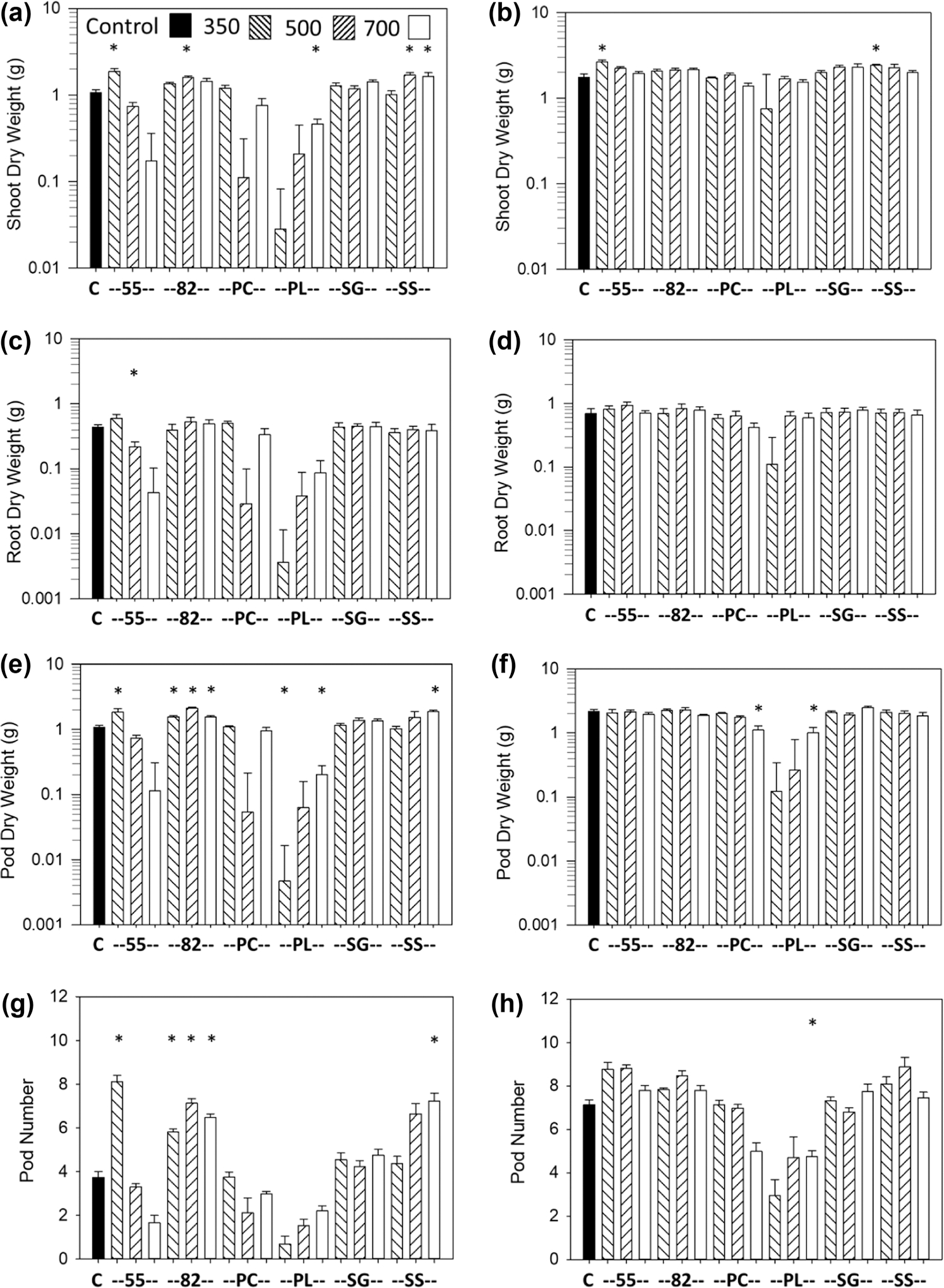
Effects of biochar on soybean dry weight and pods. Data are for shoot dry weight for (a) Norfolk or (b) Coxville soil, root dry weight for (c) Norfolk or (d) Coxville soil, pod dry weight for (e) Norfolk or (f) Coxville soil, and pod number for (g) Norfolk or (h) Coxville soil. Pyrolysis temperatures (°C) are indicated at the top of graph (a). Each bar represents the average plus the unpooled upper average SE for six pots, except for five pots for root dry weight Coxville SS 700 and four pots for the root dry weight Norfolk SS 500 and 700 treatments. An asterisk above a bar indicates a significant difference vs. control plants according to Dunnett’s test. There were no significant biochar effects on root dry weight for the Coxville soil. C, no biochar control; PC, pine chips; PL, poultry litter; SG, switchgrass; SS, swine solids; 55, 50% PC and 50% PL; 82, 80% PC and 20% PL.

The most consistent growth response was an increase in shoot and root dry weights vs. the controls with the SS biochar at all pyrolysis temperatures and with the Norfolk soil, which occurred for carrot (Figure 1a,c,e), lettuce (Figure 2a,c), and sweet corn (Figure 3a,c). Soybean also had an increase in shoot and pod dry weights and pod numbers with the SS biochar and Norfolk soil but primarily with the highest pyrolysis temperature (Figure 4a,e,g). For the Coxville soil, the SS biochar increased lettuce shoot and root dry weights (Figure 2b,d) and soybean shoot dry weight at 350 °C (Figure 4b).

**FIGURE 5:**
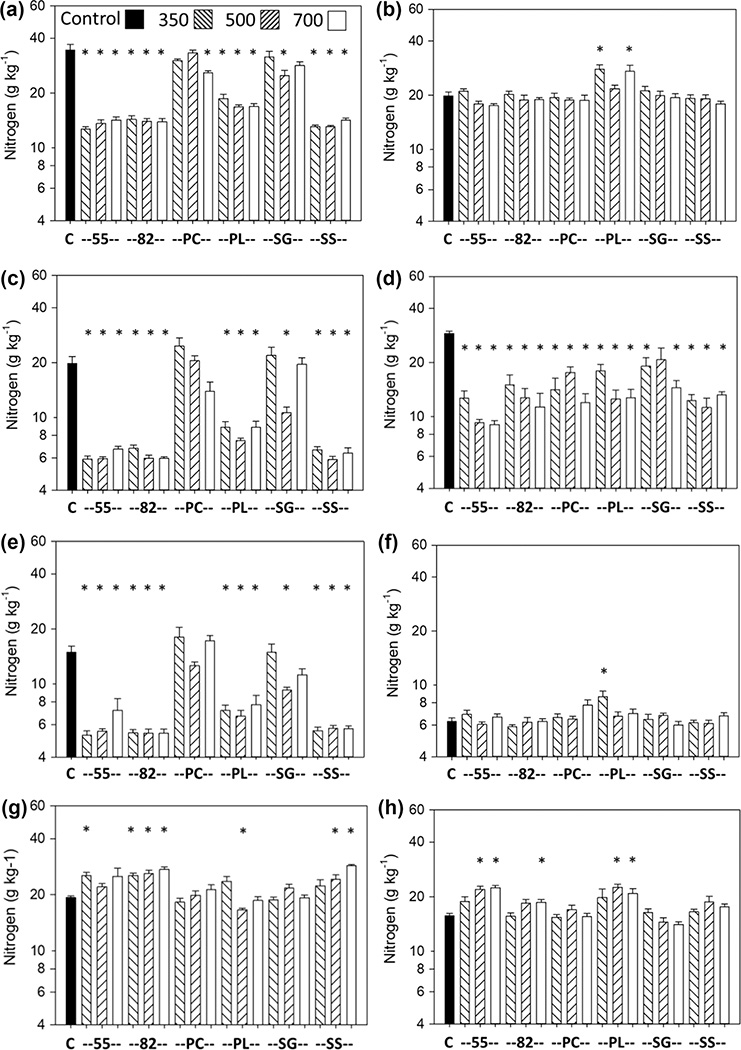
Effects of biochar on shoot (leaf or stem plus leaf for soybean) N concentration for carrot for (a) Norfolk or (b) Coxville soil, lettuce for (c) Norfolk or (d) Coxville soil, sweet corn for (e) Norfolk or (f) Coxville soil, and soybean for (g) Norfolk or (h) Coxville soil. Pyrolysis temperatures (°C) are indicated at the top of graph (a). Each bar represents the average plus the unpooled upper average SE for six pots, except for carrot there were three pots for Coxville PL 500; four for Norfolk PL 700; and five for Coxville PL 700, Norfolk PC 350 and 500, Norfolk 82 350 and 500, Norfolk PL 350 and 500 and 55 700. For lettuce there were five pots for Coxville PL 500, Norfolk PC 350, Norfolk 82 350 and 500, and Norfolk PL 500 and 700. For sweet corn there were four pots for Norfolk PL 700 and five pots for Norfolk PL 350 and 500 and SG 700. For soybean there were two pots for Norfolk PL 350; four for Norfolk PC 500; and five for Coxville PL 350, Coxville SG 500, Norfolk PL 500 and 700, and Norfolk 55 700. An asterisk above a bar indicates a significant difference vs. control plants at the .05 level according to Dunnett’s test. C, no biochar control; PC, pine chips; PL, poultry litter; SG, switchgrass; SS, swine solids; 55, 50% PC and 50% PL; 82, 80% PC and 20% PL.

The 55 biochar mixture also produced a large increase in growth for all crops, at least with some temperatures. The 55 biochar increased shoot and root dry weights with the Norfolk soil for carrot (Figure 1a,c,e), lettuce (Figure 2a,c), and sweet corn (Figure 3a,c) for at least the two lower temperatures. For the Coxville soil, the 55 biochar increased carrot taproot dry weight (Figure 1f), lettuce shoot and root dry weights (Figure 2b,d), and sweet corn shoot dry weight (Figure 3a). In contrast to the general increases in weights with the other crops, the 55 biochar both increased and decreased organ weights for soybean. The 55 biochar at 350 °C increased soybean shoot and pod dry weights, pod number for the Norfolk soil (Figure 4a,e,g), and shoot dry weight for the Coxville soil (Figure 4b). However, the 55 biochar at 350 °C decreased soybean root dry weight for the Norfolk soil (Figure 5c).

The 82 biochar mixture increased growth for each crop but not to the same extent, and effects were more variable than with the 55 or SS biochars. Results differed by crop and temperature. For the Norfolk soil, carrot shoot and root dry weights (Figure 1a,c,e) and lettuce root dry weight (Figure 2c) increased with 82 at 700 °C, and sweet corn shoot and root dry weights increased with 82 at all temperatures (Figure 3a,c). For the Norfolk soil, the 82 biochar increased soybean shoot dry weight at 500 °C and pod dry weight and number at all temperatures (Figure 4a,e,g). For the Coxville soil, the 82 biochar increased lettuce shoot and root dry weights for at least two temperatures (Figure 2b,d) and sweet corn shoot dry weight at 500 °C (Figure 3b).

In contrast to SS and the 55 and 82 mixtures, the PL-alone biochar had fewer significant effects on growth; this effect could be an increase or decrease depending on crop, temperature, and parameter. Fewer significant effects were in part due to the large SE errors for some PL treatments for all crops, especially with the Norfolk soil for all four crops (Figures 1–4), and with the Coxville soil for carrot, lettuce, and, to a lesser extent, soybean (Figures 1, 2, and 4). These large SE errors were likely related to some phytotoxicity caused by the PL biochar. For example, of the 25 pots with no plants at harvest known to be due to no germination or seedling death (out of 912 total pots across crops), 17 were for PL alone, and four were with the 55 or 82 PC and PL mixtures. In terms of significant effects, lettuce shoot and root dry weights increased with PL biochar only at 350 °C for the Norfolk soil (Figure 2a,c) but increased at 350 and 700 °C for the Coxville soil (Figure 2b,d). The PL biochar also increased sweet corn shoot dry weight with the Coxville soil (Figure 3b). In contrast, for soybean with PL at 700 °C, there were decreases in shoot and pod dry weights for the Norfolk soil and pod dry weight and number for the Coxville soil (Figure 4a,e,f,h). There also was a decrease in soybean pod dry weight with PL at 350 °C for the Norfolk soil (Figure 4e).

The PC biochar produced only a few growth responses. Lettuce with the Coxville soil had increased shoot and root dry weights at all temperatures (Figure 2b,d). Sweet corn with PC at 700 °C had decreases in shoot and root dry weights with the Norfolk soil (Figure 3a,c) and decreased root dry weight for the Coxville soil (Figure 3d). Soybean with PC and the Coxville soil had decreased pod dry weight at 700 °C (Figure 4f). The SG biochar resulted in only a few increases in growth at varying temperatures for lettuce in both soils (Figure 2a–c) and sweet corn with the Norfolk soil (Figure 3a,c).

Overall, lettuce with the Coxville soil was the most responsive to biochar, with increases in shoot dry weight with all biochars except 82 and PL at 500 °C and SG at 350 and 500 °C (Figure 2b), and in root dry weight with all biochars except for PL at 500 °C and SG at all temperatures (Figure 2d). In contrast, carrot with the Coxville soil was the least responsive to biochar, with no significant effects on shoot or fine root dry weights and only effects from the 55 biochar for taproot dry weight (Figure 1b,d,f).

### 3.3 | Crop nitrogen

There were more significant treatment effects on plant N than for growth based on the ANOVA across factors (Table 4). Significant biochar feedstock and soil effects occurred for both shoot N concentration and N uptake in all four crops. There were significant biochar pyrolysis temperature effects for all crops and N parameters except soybean shoot uptake. For most crops and N parameters, there were significant feedstock × soil, feedstock × temperature, feedstock × temperature × soil, and, to a lesser extent, temperature × soil interactions; thus, as for plant growth, data were analyzed separately for each soil on a per biochar vs. control basis.

For control plants without biochar addition, crop shoot N, and, to a lesser extent, P and K concentrations tended to be below or at the lower end of the range of sufficient concentrations for growth (Table 5), even though they received some nutrients at the time of planting. This was especially true with the Coxville soil and particularly for N in corn. Thus, the biochar treatments occurred for plants under some nutrition stress, as discussed below.

**FIGURE 6:**
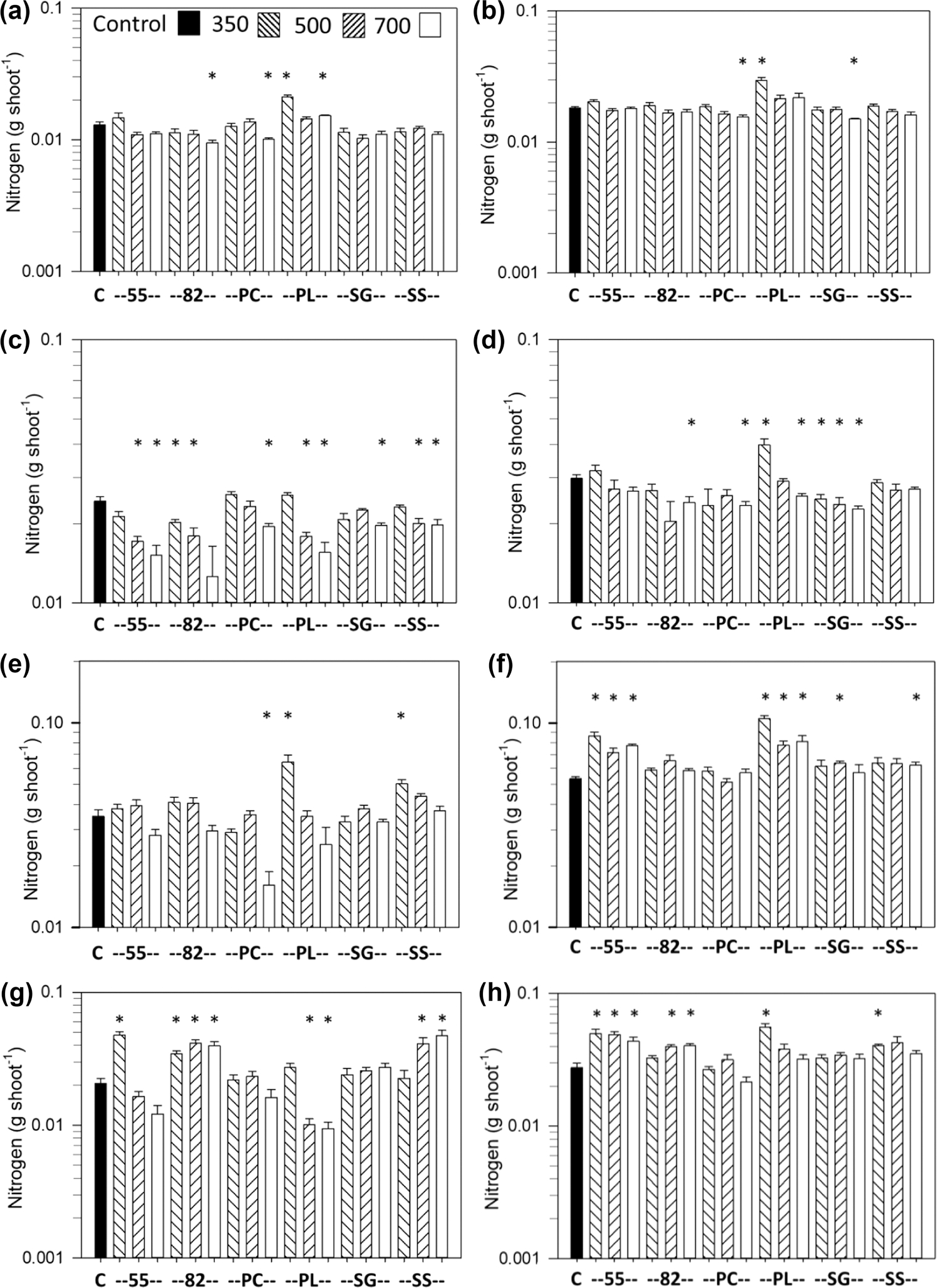
Effects of biochar on shoot (leaf or stem plus leaf for soybean) N uptake for carrot for (a) Norfolk or (b) Coxville soil, for lettuce for (c) Norfolk or (d) Coxville soil, for sweet corn for (e) Norfolk or (f) Coxville soil, and for soybean for (g) Norfolk or (h) Coxville soil. Pyrolysis temperatures (°C) are indicated at the top of graph (a). Each bar represents the average plus the unpooled upper average SE for six pots, except for carrot there were three pots for Coxville PL 500; four for Norfolk PL 700; and five for Coxville PL 700, Norfolk PC 350, Norfolk 82 350 and 500, Norfolk PL 350 and 500, and 55 700. For lettuce there were five pots for Coxville PL 500, Norfolk PC 350, Norfolk 82 350 and 500, and Norfolk PL 500 and 700. For sweet corn there were four pots for Norfolk PL 700 and five for Norfolk PL 350 and 500, SG 700, and the control. For soybean there were two pots for Norfolk PL 350; four for Norfolk PC 500; and five for Coxville PL 350, Coxville SG 500, Norfolk PL 500 and 700, and Norfolk 55 700. An asterisk above a bar indicates a significant difference vs. control plants at the .05 level according to Dunnett’s test. C, no biochar control; PC, pine chips; PL, poultry litter; SG, switchgrass; SS, swine solids; 55, 50% PC and 50% PL; 82, 80% PC and 20% PL.

Based on comparison to the controls using Dunnett’s test, the biochars that had the greatest effects on plant growth also dramatically affected shoot nutrient concentrations. The differences in direction of N response were highly dependent on crop and soil (Figures 5,6). The SS, 55, 82, and PL biochars decreased shoot N concentration at all temperatures with the Norfolk soil for carrot (Figure 5a), lettuce (Figure 5c), and sweet corn (Figure 5e) and with the Coxville soil for lettuce (Figure 5d). The PL biochar also decreased shoot N concentration for soybean with the Norfolk soil at 500 °C (Figure 5g). The SG biochar decreased shoot N concentration with the Norfolk soil at 500 °C for carrot (Figure 5a), lettuce (Figure 5c), and sweet corn (Figure 5e). The SG biochar also decreased shoot N concentration with the Coxville soil for lettuce at 350 and 700 °C (Figure 5d). The PC biochar decreased shoot N concentration for carrot with the Norfolk soil at 700 °C (Figure 5a) and for lettuce with the Coxville soil at all temperatures (Figure 5d).

In contrast, a few biochar treatments increased shoot N concentration. The PL biochar increased the shoot N concentration with the Coxville soil for carrot at 350 and 700 °C (Figure 5b) and sweet corn at 350 °C (Figure 5f). For soybean and the Norfolk soil, the SS, 55, and 82 biochars increased shoot N concentration for at least one pyrolysis temperature (Figure 5g). For soybean with the Coxville soil, the 55, 82, and PL biochars increased shoot N concentration for at least one temperature (Figure 5h).

When shoot N was expressed on an uptake basis (g shoot^−1^), many of the differences between biochar treatments and the controls were smaller or different than when expressed as shoot N concentration. The differences between shoot N uptake and N concentration were most dramatic for carrot and sweet corn with the Norfolk soil (Figure 6a,e), for lettuce with the Coxville soil (Figure 6d) where there were fewer biochar effects on N uptake, and for sweet corn with the Coxville soil where there were more biochar effects on N uptake (Figure 6f). For carrot and the Norfolk soil, the only decreases in N uptake were with 82 and PC at 700 °C PL, whereas N uptake increased with PL at 350 and 700 °C (Figure 6a). For sweet corn and the Norfolk soil, N uptake decreased with only PC at 700 °C but increased with PL and SS at 350 °C (Figure 6e). Lettuce and the Coxville soil had decreases in N uptake for SG at all temperatures and 82, PL, and PC at 700 °C, whereas PL at 350 °C had an increase in N uptake (Figure 6d). Sweet corn with the Coxville soil had increases in N uptake with 55 and PL at all temperatures as well as SS at 700 °C and SG at 500 °C (Figure 6f). In contrast, for carrot with the Coxville soil (Figure 6b), lettuce with the Norfolk soil (Figure 6c), and soybean with both soils (Figure 6g,h), the pattern for changes in N uptake was somewhat like the pattern for shoot N concentration across biochar treatments.

## 4 | DISCUSSION

### 4.1 | Effects of biochar on crop growth

These results indicate that the addition of biochar to coastal plain soils in the southeastern United States would likely enhance plant growth. However, the biochar used should be carefully designed to address specific soil quality characteristics (Novak et al., 2014). Despite the uniform biochar treatments, soils, and environmental conditions in our study, each of the four crops responded somewhat differently, reflecting the range of crop responses previously reported for biochar in the literature (Biederman & Harpole, 2013; Jeffery et al., 2011, 2017; Liu et al., 2013).

Our results supported the conclusions that there are benefits to soils (Novak & Busscher, 2012) and crops (Sigua, Novak, Watts, Johnson, & Spokas, 2016) from soil addition of designer biochars made from blends of specific feedstocks. In this study, SS and 55 (a mixture of a manure-based biochar [PL] and a cellulosic biochar [PC]) most consistently enhanced the growth of lettuce, carrot, and sweet corn. The growth enhancement with 55 was greater than with PL or PC alone. Previously Sigua et al. (2016) reported that the same 55 and 82 biochar mixtures that we used resulted in increases in aboveground wheat biomass compared with the no-biochar control and that the increases were similar to those with the PC biochar alone. The 55 and 82 aboveground and belowground biomass amounts were much greater than with the PL biochar alone, and the PL biomass was dramatically reduced compared with the control (Sigua et al., 2016). Although PL biochar can act as a fertilizer by supplying nutrients (e.g., N, P, K) (Novak & Busscher, 2012), it also had the highest EC and ash contents and a relatively higher Na concentration (Table 2). High Na and other nutrients in biochar can have a negative effect on crop growth (Sigua et al., 2016; Subedi et al., 2017). Addition of the PC biochar to PL in the mixture likely alleviated some of the problems with PL, such as the high Na contents, while improving other soil characteristics, such as water-holding capacity (Novak & Busscher, 2012).

The greater impact on crop growth with manure-based (primarily with SS, 55, and 82) and only slightly with PL biochars than with a cellulose-based (PC and SG) biochars was similar to findings reported previously (Jeffery et al., 2011; Liu et al., 2013). Here, increased growth with these manure-based biochars was observed across carrots, lettuce, and sweet corn, especially for the Norfolk soil. Even though significant growth increases were more limited for soybean, those that did occur tended to be with SS as well as 55 and 82 biochars and for the Norfolk soil. Enhanced growth with the manure biochars was likely related to their ability to supply necessary plant nutrients (Hass et al., 2012). These characteristics would be more important in the sandy-textured and relatively more nutrient-poor Norfolk soil (Table 1).

Our study confirmed the conclusions of Domingues et al. (2017) and Rajkovich et al. (2012) that the biochar feedstock was more important than pyrolysis temperature in determining biochar characteristics and ecological effects. For example, separate from the many interactions among factors and across crops, feedstock by itself was significant across temperatures and soils for 10 of the 11 growth parameters, whereas temperature by itself was significant for one growth parameter (Table 4). Similarly, Bonanomi et al. (2017) showed larger differences in lettuce growth among nine types of biochars than between two pyrolysis temperatures.

Our finding that the sandier Norfolk soil had increased crop growth when compared with the Coxville soil for many measurements when biochar was added for carrot, sweet corn, and soybean was similar to Liu et al. (2013), who reported that crop productivity was greater in sandy soils following the addition of biochar compared with clay, loam, or silt soils. In an example of a similar result in a specific study, Aller, Rathke, Laird, Cruse, and Hatfield (2017) reported greater fresh maize biomass with addition of fresh biochar to sandy and silt loam soils compared with decreased biomass with a clay loam soil, whereas aged biochar increased fresh biomass only in the silt loam soil. However, there were exceptions, such as decreased sweet corn shoot biomass with biochar in a sandy soil but increased shoot biomass with a clay soil, which was attributed to the higher concentrations of ions such as OH^−^ and soluble salts from the biochar coupled with the lower buffering capacity of the sandy soil (Smider & Singh, 2014). The greater increases in crop growth with biochar addition for the sandy Norfolk soil in our study may be related to its lower inherent fertility (e.g., negligible N, lower soil organic C, and lower P) and therefore that greater benefits were derived from the biochar addition, even though both soils received the same supplemental fertilizer treatments. This may be related to greater nutrient accessibility in the Norfolk soil than in the Coxville soil due to subtle differences in soil texture and mineralogy (Table 1).

Overall, lettuce was the most responsive crop to biochar amendment, with increases in dry weight with each feedstock for at least one pyrolysis temperature and soil type (Figure 2). As in other recent studies, we found increases in lettuce growth with many feedstocks, not only manure-based, but also cellulosic. For example, Anderson et al. (2017) found increases in lettuce shoot and root biomass with walnut shell biochar, but Gartler et al. (2013) reported no effects of *Pinus radiata* D. Don chip biochar on lettuce shoot growth. Artiola, Rasmussen, and Freitas (2012) reported that biochar produced from pine waste material initially had no effect or decreased lettuce head wet weights but subsequently increased weights after a period of adaptation of biochar to the pots and modification of the fertilizer and/or watering regimes. Similar to our results with lettuce, Bonanomi et al. (2017) found that an organic waste biochar (municipal solid waste) increased lettuce growth the most; however, in contrast to our results, they reported that a more cellulosic *Zea mays* stalk biochar actually decreased lettuce growth the most.

In terms of specific crop responses, sweet corn showed a range of growth responses to biochar (Figure 3), as found in other specific studies and in a meta-analysis for both sweet corn and maize in general (Brantley et al., 2015; Butnan et al., 2015; Cole et al., 2019; Haider et al., 2015; Jeffery et al., 2015; Smider & Singh, 2014). Our reported responses ranged from the largest significant decrease (based on least square means) in sweet corn shoot dry weight with the Norfolk soil and PL at 700 °C (107%) to the largest increase with the Norfolk soil and SS at 350 °C (167%), based on means for the Dunnett’s test. Over both soils and all biochars, shoot dry weight increased by an average of 28%, compared with a 19% increase in maize yield (Jeffery et al., 2015) and a <10% increase maize biomass and 10–20% increase in maize yield (Liu et al., 2013), across a variety of studies. This difference may be due in part to the fact that pot studies have been observed to have larger increases in crop growth responses than field studies (Liu et al., 2013).

For the other crops, specific growth responses also fell within the wide range of those reported in the literature. For example, we found increases in carrot biomass primarily with PL and 55 but not with biochar produced from cellulosic feedstocks. Previous studies with cellulosic feedstocks also found no, or few, effects of biochar on carrots. For example, carrot root growth was not affected by *Pinus radiata* D. Don chip biochar (Gartler et al., 2013). Carrot shoot, tap root, and fine root biomass were not affected by most pine wood, pine bark, wood pellet, or spelt husk biochar treatments; the only significant effects were increases in taproot biomass with wood pellet and spelt husk biochars (George et al., 2016). Anderson et al. (2017) reported no effects on carrot shoot or root growth from walnut shell biochar at a rate of 10 Mg ha^−1^ and that only a very high biochar rate of 100 Mg ha^−1^ may have increased carrot root growth.

The minimal effects of biochar on soybean growth in our study were as found in some other studies. For example, Egamberdieva, Wirth, Behrendt, Abd_Allah, and Berg (2016) reported that neither maize nor wood biochar affected soybean root or shoot dry weight. Backer, Schwinghamer, Whalen, Sequin, and Smith (2016) reported that pine wood biochar did not alter soybean seed yield in a field study. Yu et al. (2017) found an increase in soybean yield and seed biomass with added biochar when plants also received urea or ammonium sulfate fertilizer but not with potassium nitrate fertilizer.

In our biochar treatments where there was no increase in plant growth, the lack of growth may be because nutrients were limited, as shown for the no-biochar controls vs. reference leaf nutrient sufficiency concentration ranges (Table 5), even though we applied P and K fertilizer to all crops and N to carrot, lettuce, and sweet corn. Although our control carrot shoot N, P, and K concentrations were similar to reference leaf levels, lettuce shoot concentrations were slightly lower than reference leaf levels, and soybean and especially sweet corn shoot concentrations with the Coxville soil were considerably lower than reference leaf concentrations. Overall, shoot N, P, and K concentrations were lower with the Coxville than with the Norfolk soil. The lowest elemental concentration for control plant vs. reference leaves was for sweet corn shoot N with the Coxville soil, where the 5–7 g kg^−1^ (Table 5) is far below the <17.5 g kg^−1^ considered to be deficient for maize leaves (ear leaf tissue at silking to tasseling; Schulte & Kelling, 1991). An example of the importance of fertilization to maximize biochar effects was reported by Deenik and Cooney (2016), who found large increases in maize shoot dry weight with biochar only when it was combined with fertilized soil for corn cob biochar across three cropping cycles and sewage sludge biochar across two cycles. For one cropping cycle, the sewage sludge biochar alone increased maize shoot dry weight, but the increase was much greater when the soil also was fertilized (Deenik & Cooney, 2016). Furthermore, the importance of adequate fertilizer for biochar studies was shown in a study with sweet corn in the field, where Cole et al. (2019) found that biochar reduced yield and stalk nitrate-N and noted that the fertilizer was less than the recommended rate in New England. In addition, based on a review of the relationship between crop responses to biochar and N and P cycling, Gul and Whalen (2016), concluded that inorganic fertilizer is needed for biochar to improve crop performance.

### 4.2 | Effects of biochar on shoot nitrogen

Successful plant growth depends on an adequate supply of plant nutrients, with growth rates dependent on the availability of essential nutrients, such as N (McDonald, 1994). Enhanced growth with biochar treatments containing animal manure (55, 82, SS, and PL) was likely due to a N-enhancing (Clough et al., 2013) and a general nutrient-enhancing effect (Agegnehu et al., 2017) because the initial soil levels of N, P, and K appeared to be low (Table 1) and the levels of these elements high in the at least partially manure-based biochars (Table 2).

However, the decreases in shoot nutrient concentrations with many biochar treatments may be predictive of eventual growth reductions because the concentrations generally were below nutrient sufficiency concentration ranges for these crops even without the addition of biochar (Table 5) (Hanlon & Hochmuth, 2000; Hartz, Johnstone, Williams, & Smith, 2007; MacKay & Leefe, 1962; Mueller, 2019). Decreases in shoot N were more significant for the manure-based biochars (SS and PL) and blends (55 and 82) than for the cellulosic biochars. This was similar to the greater decreases in lettuce shoot Ca, Mg, and Zn seen with manure-based biochars compared with cellulosic biochars for the same plants (Olszyk et al., 2020). Shoot levels of N also, in general, decreased more in the crops that received N fertilizer (carrot, lettuce, sweet corn) than for soybean, which did receive N fertilizer but which relied on N fixation. In carrot and sweet corn, decreases in shoot N concentration generally were more pronounced with the sandier and lower N content Norfolk than with the Coxville soil (Figure 5a,b and e,f).

Greater plant N decreases with a sandier soil such as the Norfolk soil than with the less sandy Coxville soil were suggested in other studies. For example, Syuhada, Shamshuddin, Fauziah, Rosenani, and Arifin (2016) found a reduction in maize shoot N concentration when fertilizer was added with biochar with a fertilized sandy Humo-Ferric Podzol, and Haider et al. (2015) reported a reduction in maize leaf N with biochar added to a sandy soil. In contrast, Gaskin et al. (2010) found no effect of peanut hull or pine chip biochar on maize leaf N for plants growing on a loamy sand Ultisol.

Our shoot N results highlighted the complexity of the biochar and soil N relationship as reviewed by Clough et al. (2013) and Liu et al. (2018). Decreases in shoot N following the application of biochar have been observed in other studies with the species we studied. For example, sweet corn stalk nitrate-N decreased with the addition of sugar maple wood trimming biochar, especially for unfertilized plants but also for fertilized plants that received less than the recommend fertilizer rate (Cole et al., 2019). Syuhada et al. (2016) observed a reduction in maize shoot N concentration with the addition of oil palm biochar to a fertilized soil but observed no effect of biochar on shoot N in unfertilized soil; however, shoot N uptake increased with biochar, especially for the fertilized soil. Our general decrease in shoot N was in contrast to the only slight reduction (2%) in plant tissue N concentration but an increase (11%) in plant N uptake reported by Liu et al. (2018).

Syuhada et al. (2016) suggested that a decrease in shoot N in maize with biochar was related to growth dilution (Jarrell & Beverly, 1981), meaning the plant tissue nutrient content was decreased with an increase in plant dry matter. Another example of a decrease in aboveground leaf N concentration but an increase in aboveground biomass with biochar was reported for maize by Haider et al. (2015). Lehmann et al. (2003) found a decrease in aboveground shoot N concentration with a variable, nonsignificant decrease in N uptake but an increase in cowpea aboveground biomass with charcoal (not biochar). In contrast, some plants showed the opposite of a dilution effect with biochar. For example, fertilized (but not unfertilized) radish plants with biochar had a large increase in N uptake and no effect on N concentration with a large increase in biomass (Chan, Van Zwieten, Meszaros, Downie, & Joseph, 2007). Rogovska, Laird, Rathke, and Karlen (2014) reported no effect of biochar on stalk N concentration, even though there was an increase in shoot biomass for one of two growing seasons.

Factors other than growth dilution are likely associated with decreases in shoot N. For example, O’Toole, Knoth de Zarruk, Steffens, and Rasse (2013) reported a reduction in leaf N concentration but little effect on ryegrass biomass with wheat straw biochar, which they attributed to decreases in soil N availability due to adsorption of N onto biochar surfaces and increased microbial N fixation. Kim et al. (2015) indicated that high application rates of a cellulosic biochar (rice hull feedstock) resulted in decreased lettuce biomass and reduced soil inorganic N, which was attributed to adsorption of N onto biochar. In contrast to adverse effects on some crops, Hagemann, Kammann, Schmidt, Kappler, and Behrens (2017) suggested that biochar-induced retention and the slow release of nitrate from soils biochar could have environmental benefits. Thus, to better understand the dynamics of soil N that affect crop growth, research is needed on the relationship between biochar on N capture and release (Hagemann et al., 2017) and N transformations in soil (DeLuca, Gundale, MacKenzie, & Jones, 2015).

The unique N response for soybean in our study (primarily increases in both shoot N concentration and uptake) likely was related to the effect of biochar on N fixation because this crop received no N fertilizer at planting but was inoculated with N-fixing bacteria. Biochar tended to increase microbial activity for soybean, even though there was a decrease in microbial activity for a soil × fertilizer combination (Van Zwieten et al., 2010). Scheifele et al. (2017) reported increased soybean root nodule number and dry matter, shoot N content, and plant biomass content across four soils from maize biochar, but with wood biochar they reported only increasing shoot N content. In another legume (red clover, *Trifolium pratense* L.), 10 Mg ha^−1^ of grassland biochar increased biological N fixation and aboveground biomass, with generally adverse effects as the biochar rate increased to 120 Mg ha^−1^ (Mia et al., 2014). Common bean showed increases in biological N fixation with the addition of *Eucalyptus deglupta* Blume log biochar, which was attributed primarily to increased B and Mo availability (Rondon, Lehmann, Ramirez, & Hurtado, 2007).

### 4.3 | Designer biochars

This study clearly indicated the variability in crop responses to biochar and that no single biochar type was effective for crop growth improvements. We also showed that different biochars can have variable impacts on crop N status. The literature has reported that not all biochars can improve crop yields (Spokas et al., 2012), so the counter paradigm is to produce designer biochar using specific feedstocks and/or modifying pyrolysis conditions (Novak & Busscher, 2012). In this way, designer biochars will have certain characteristics that can be tailored to address specific soil deficiencies (e.g., nutrients, labile C, water-holding capacity). Doing so avoids using the wrong biochar on the wrong soil because biochars cannot be removed from a field after application. Making a designer biochar will require careful modifications of pyrolysis conditions. The complexity of this process can be reduced by improved communication between soil scientists, biochar producers, and thermal process engineers. For example, if a designer biochar is selected to raise soil pH to a given level (i.e., pH 6–7), this would improve soil pH ranges, which benefits plants accustomed to that range. Additionally, if a designer biochar was selected to supply micronutrients or N to soil, then this action would benefit all plants grown within that specific crop rotation.

## 5 | CONCLUSIONS

With consistent biochar treatments, soils, and growing conditions, crops still varied in response but with some general patterns. Although there were some differences in response due to biochar pyrolysis temperature, in general, temperature had less effect then feedstock on the crop responses. As suggested by the literature, manure-based biochars (i.e., SS and 55 blend of PL and PC) were the most effective in increasing shoot and root biomass for carrots, lettuce, and sweet corn, especially when applied to a more coarse-textured sandy Norfolk soil than to the finer-textured Coxville soil for carrot and sweet corn. Results were somewhat similar for soybean and the Norfolk soil, where some biochars made from SS and 55 blends of PL and PC increased shoot and pod dry weights. However, for soybean a few biochars made from PL alone and the PL/PC blends decreased shoot, root, or pod biomass. The SS, PL, and blends of PC and PL biochars tended to decrease shoot N concentration for lettuce, carrot, and sweet corn, especially with the Norfolk soil, although there were a few cases of increases in N. Some PC and SG biochars also decreased shoot N concentration, but with a few increases. In some cases (i.e., carrot and sweet corn with Norfolk soil), when N was expressed on a shoot uptake basis, the decreases in shoot N concentration did not occur, suggesting nutrient dilution resulting from increased growth. However, in other cases, especially for lettuce, there were similar decreases in N expressed as shoot N concentration or N uptake for many biochar treatments, indicating other reasons for decreased N in the plants. Because of the potential for biochar to reduce concentrations of the important plant nutrient N, the biochar used must be carefully considered for a crop to minimize any decreases in N while increasing crop growth. Specific biochar feedstock type and pyrolysis temperatures, as well as soil characteristics and crop, must all be considered to optimize use of biochar as a soil amendment.

## Figures and Tables

**TABLE 1:** Chemical characteristics of Coxville and Norfolk soils

Soil	pH^a^	Sand	Silt	Clay	Organic C	Total N	K	P	Ca	Mg	Zn	Cu	Mn	B	Na
		g k^−1^					mg kg^−1^								
Coxville	5.1	421	434	145	26.3	1.8	40	44	321	53	3.0	0.8	10	0.2	8
Norfolk	5.9	807	167	26	3.9	ND^b^	53	17	257	35	3.7	0.5	6	0.1	4

*Note.* Adapted from Sigua et al. (2014), Olszyk et al. (2018, 2020). The K, P, Ca, Mg, Zn, Cu, Mn, B, and Na data are based on a Mehlich 1 extraction solution (J. Novak, personal communication, 2018).

a Soil/water ratio of 1:2.

b Not detected (i.e., below detection limit of 1 g kg^−1^).

**TABLE 2:** Key chemical characteristics of biochars used in this study based on a variety of sources^a^

Feedstock^b^	Temperature	pH	EC	Ash	C	H	O	N	K	Ca	Mg	Na	S	P	Zn	EP
	°C		mS cm^−1^	g kg^−1^												mg L^−1^
PL	350	8.73	16.4	359^c^	461^c^	37^c^	86^c^	50^c^	56	35	12	16.0	9.8	25	0.9	81.3
PL	500	9.76	18.9	409^f^	483^f^	15^f^	46^f^	39^f^	70	47	17	22.5	11.6	34	1.2	61.0
PL	700	10.30	20.4	524^c^	440^c^	3^c^	0.1^c^	28^c^	77	50	18	24.8	9.8	35	1.2	16.4
PC/PL 55	350	7.68	8.59	185^d^	637^d^	38^e^	102^e^	34^d^	34	21	8	10.2	5.0	13	0.6	201.4
PC/PL 55	500	9.99	8.99	222^f^	694^f^	18^f^	37^f^	24^f^	36	26	9	11.2	4.8	18	0.6	100.1
PC/PL 55	700	10.44	9.92	248^f^	712^f^	7^f^	10^f^	17^f^	43	27	9	11.2	3.0	18	0.5	67.4
PC/PL 82	350	7.69	2.54	73^d^	758^d^	46^e^	108^e^	13^d^	11	9	3	2.9	1.5	5	0.2	195.0
PC/PL 82	500	9.66	3.04	92^f^	836^f^	27^f^	32^f^	13^f^	16	12	4	4.1	1.4	6	0.3	104.2
PC/PL 82	700	10.08	3.78	101^f^	886^f^	10^f^	<0.1^f^	10^f^	14	10	3	4.3	<0.1	5	0.1	60.3
SS	350	6.94	3.14	350^e^	510^e^	37^e^	32^e^	59^e^	13	37	31	4.3	10.8	50	5.0	181.4
SS	500	7.80	2.98	438^f^	451^f^	15^f^	30^f^	55^f^	21	53	42	6.0	10.1	>50	6.8	195.2
SS	700	8.74	1.64	488^f^	454^f^	5^f^	5^f^	36^f^	21	56	44	6.4	7.2	>50	6.8	136.8
PC	350	5.74	0.37	15^c^	747^c^	50^c^	184^c^	5^c^	2	3	0.9	0.1	<0.1	0.3	0.04	7.2
PC	500	7.57	0.42	23^c^	872^c^	36^c^	65^c^	4^c^	3	5	1.2	0.1	<0.1	0.6	0.08	3.6
PC	700	8.92	0.51	29^f^	966^f^	12^f^	<0.1^f^	4^f^	2	3	0.4	0.1	<0.1	0.2	0.03	0.04
SG	350	5.76	0.33	32^e^	755^e^	46^e^	162^e^	5^e^	2	3	1.9	0.1	0.7	0.8	0.03	13.6
SG	500	8.38	0.79	78^c^	844^c^	24^c^	43^c^	11^c^	4	4	2.5	0.1	0.7	1.5	0.06	44.7
SG	700	9.56	0.80	55^f^	941^f^	12^f^	<0.1^f^	5^f^	4	3	1.0	0.1	<0.1	0.6	0.02	30.2

*Note.* Total K, Ca, Mg, Na, S, P, and Zn concentrations are based on analysis of one sample by a commercial laboratory. EC, electrical conductivity; EP, extractable phosphorus. Except for EP all element amounts are totals. Values are on a % dry-weight basis except for pH (as H_2_O), EC, and EP.

a Adapted from Olszyk et al. (2018, 2020) if no other superscript.

b PC, pine chips; PL, poultry litter; SG, switchgrass; SS, swine solids; 55, 50% PC and 50% PL; 82, 80% PC and 20% PL.

c Adapted from Novak et al. (2013).

d Adapted from Novak et al. (2014).

e Adapted from Novak & Johnson (2019).

f From J. Novak (personal communication, 2016, 2020).

**TABLE 3:** Key cultural dates and environmental conditions for four crops treated with different biochars

Crop	Planted	Emerged	Harvested	Avg^a^ min. temp.	Avg. max. temp.	Avg. daily temp.	Avg. PAR^b^	Avg. RH^c^	Avg. CO_2_
				°C			μmol m^−2^ s^−1^	%	ppm
Sweet corn	15 Aug. 2013	20 Aug. 2013	18 Oct. 2013	21.3	25.4	18.1	165	61	424
Soybean	16 Aug. 2013	20 Aug. 2013	19 Oct. 2013	21.3	25.4	18.0	166	61	425
Lettuce	8 Nov. 2013	10 Nov. 2013	15 Jan. 2014	19.0	22.4	15.3	188	39	442
Carrot	13 Nov. 2013	17 Nov. 2013	22 Jan. 2014	19.2	22.7	15.3	195	36	440

a Each average is the average of hourly values for the growth period for each species.

b Photosynthetically active radiation between 400 and 700 nm.

c Relative humidity.

**TABLE 4:** Results from ANOVA for effects of biochar treatments (soil [S], feedstock [F], temperature [T]) on crop growth and nitrogen

Crop	Response	*n*	*P* values						
			S	F	T	F × S	T × S	F × T	F × T × S
Carrot	shoot dry weight	216	<.**001**	.120	.338	.138	<.**001**	.501	.432
Carrot	diffuse root dry weight	216	**.011**	<.**001**	.631	<.**001**	.264	.217	.404
Carrot	tap root dry weight	216	.120	<.**001**	.716	<.**001**	.239	.140	**.011**
Carrot	shoot N, g kg^−1^	203	<.**001**	<.**001**	**.001**	<.**001**	.223	**.007**	<.**001**
Carrot	shoot N, g shoot^−1^	203	<.**001**	<.**001**	<.**001**	.074	.679	<.**001**	**.013**
Lettuce	shoot dry weight	215	.091	<.**001**	.833	<.**001**	.770	**.005**	**.001**
Lettuce	root dry weight	214	**.008**	<.**001**	.792	<.**001**	.907	.161	.107
Lettuce	shoot N, g kg^−1^	210	<.**001**	<.**001**	<.**001**	<.**001**	**.008**	**.003**	<.**001**
Lettuce	shoot N, g shoot^−1^	210	<.**001**	<.**001**	<.**001**	<.**001**	**.034**	<.**001**	.636
Soybean	shoot dry weight	216	<.**001**	<.**001**	.905	**.001**	.606	<.**001**	<.**001**
Soybean	root dry weight	211	<.**001**	<.**001**	.760	**.012**	.482	**.007**	**.046**
Soybean	pod dry weight	216	<.**001**	<.**001**	.413	<.**001**	.914	<.**001**	<.**001**
Soybean	pod number	216	<.**001**	<.**001**	.653	<.**001**	.497	<.**001**	<.**001**
Soybean	shoot N, g kg^−1^	205	<.**001**	<.**001**	.088	<.**001**	**.012**	**.008**	<.**001**
Soybean	shoot N, g shoot^−1^	205	<.**001**	<.**001**	<.**001**	<.**001**	**.009**	<.**001**	<.**001**
Sweet corn	shoot dry weight	216	<.**001**	<.**001**	**.012**	<.**001**	**.040**	<.**001**	<.**001**
Sweet corn	root dry weight	216	<.**001**	<.**001**	.235	<.**001**	.328	<.**001**	**.030**
Sweet corn	shoot N, g kg^−1^	211	<.**001**	<.**001**	<.**001**	<.**001**	.067	**.004**	<.**001**
Sweet corn	shoot N, g shoot^−1^	211	<.**001**	<.**001**	<.**001**	<.**001**	<.**001**	<.**001**	<.**001**

*Note.* There were a possible 216 values per analysis (2 soils × 18 biochar treatments × 6 replicates). Values in bold are significant at p < .05.

**TABLE 5:** Range of elemental concentrations for six control shoots (all leaf except for some stem for soybean) samples from Coxville and Norfolk soils and examples of reference leaf nutrient concentration ranges

	Range of control concentrations									
	N		P		K		Reference concentrations			
					
Crop	Coxville	Norfolk	Coxville	Norfolk	Coxville	Norfolk	N	P	K	Reference
	g kg^−1^									
Carrot	17–25	29–49	1.6–2.0	3.5–4.5	21–26	33–39	18–25	2–4	20–40	Hanlon & Hochmuth (2000); 60 d after seeding
Lettuce	25–31	15–31	2.2–3.1	2.7–4.2	17–23	15–24	43–56	4.5–7.5	33–64	Hartz et al. (2007); early heading, optimum range by Diagnosis and Recommendation Integrated System Analysis
Soybean	14–17	18–22	0.7–1.1	2.1–3.6	7–9	12–15	45–60	3.5–5.5	20–30	Mueller (2019); sufficiency range
Sweet corn	5–7	13–23	1.2–1.4	3.4–5.1	7–9	18–30	35–38	3.4–4.1	21–31	MacKay & Leefe (1962); optimum levels for sweet corn across three stages of development

## Core Ideas

- Effects of a variety of biochars with two soils on four crops were evaluated in a greenhouse study.
- Growth and N responses differed with crop, soil, and biochar feedstock and pyrolysis temperature.
- Growth increases predominated, especially with the sandier soil and manure-based biochars.
- Biochar tended to decrease carrot, lettuce, and sweet corn shoot N concentration, but N uptake varied.
- Soybean shoot N concentration and uptake primarily increased with biochar amendments.

## Abbreviations:

PC: pine chips
PL: poultry litter
RO: reverse osmosis
SG: switchgrass
SS: swine solids
55: 50% pine chips and 50% poultry litter
82: 80% pine chips and 20% poultry litter
